# Harnessing the Potential of Stem Cells for Disease Modeling: Progress and Promises

**DOI:** 10.3390/jpm10010008

**Published:** 2020-02-06

**Authors:** Chiara Argentati, Ilaria Tortorella, Martina Bazzucchi, Francesco Morena, Sabata Martino

**Affiliations:** 1 Department of Chemistry, Biology and Biotechnologies, University of Perugia, Via del Giochetto, 06126 Perugia, Italy; chiara.argentati89@gmail.com (C.A.); tortorella.i@hotmail.it (I.T.); martina.bazzucchi89@gmail.com (M.B.); francesco.morena@unipg.it (F.M.); 2 CEMIN, Center of Excellence on Nanostructured Innovative Materials, Via del Giochetto, 06126 Perugia, Italy

**Keywords:** organoids, organ-on-a-chip, iPSCs, gene therapy, genome editing, tissue engineering, computational cell modeling

## Abstract

Ex vivo cell/tissue-based models are an essential step in the workflow of pathophysiology studies, assay development, disease modeling, drug discovery, and development of personalized therapeutic strategies. For these purposes, both scientific and pharmaceutical research have adopted ex vivo stem cell models because of their better predictive power. As matter of a fact, the advancing in isolation and in vitro expansion protocols for culturing autologous human stem cells, and the standardization of methods for generating patient-derived induced pluripotent stem cells has made feasible to generate and investigate human cellular disease models with even greater speed and efficiency. Furthermore, the potential of stem cells on generating more complex systems, such as scaffold-cell models, organoids, or organ-on-a-chip, allowed to overcome the limitations of the two-dimensional culture systems as well as to better mimic tissues structures and functions. Finally, the advent of genome-editing/gene therapy technologies had a great impact on the generation of more proficient stem cell-disease models and on establishing an effective therapeutic treatment. In this review, we discuss important breakthroughs of stem cell-based models highlighting current directions, advantages, and limitations and point out the need to combine experimental biology with computational tools able to describe complex biological systems and deliver results or predictions in the context of personalized medicine.

## 1. Introduction

Over the past decades, much of our understanding of human physiology and pathologies has been derived from studies on animal models. However, during the years, emerging limitations have raised doubts about the reliability of this investigational approach [1]. The main criticisms about the actual predictive value of animal models are related to (i) their limits on fully recapitulating the human physiology/pathologies (e.g., different pharmacokinetics and toxicokinetic; presence of alternative pathways that may interfere with the progression of a given disease; different immune-response), (ii) their maintenance costs, and (iii) the ethical issues associated with their use [2,3,4]. Thus, even if animal models, especially mammals, are still an essential tool before a novel therapeutic strategy enters clinical trials, their use is severely restricted [5]. In order to overcome these limitations, scientists have focused on finding alternative approaches and tools that could recapitulate the homeostasis of cells/tissues as well as pathological alterations. Therefore, in vitro cell/tissue models today became a very promising tool for investigating human development, physiology and disease pathogenesis.

Here, we review the progress made in establishing the most proficient cell/tissue modeling, from basic cell culture systems to more innovative organoid platforms.

## 2. Cell Modeling Overview: From Basic 2D-Cultures to 3D-Innovative Systems

Since their establishment, cell cultures have been proven to be the first and most powerful tool for the in vitro investigation of cell biology, from basic research to more complex translational approaches [6]. Classical two-dimensional (2D)-cultures usually consist of a unique type of cells (primary or continuous cultures) that grow in tissue culture polystyrene as adherent monolayers or in floating suspension, both in culture media that provide essential nutrients and growth factors. 2D-strategies are widely used to obtain a large number of cells, thus allowing the in vitro study of molecular mechanisms and development of metabolic assays [7,8,9]. However, due to their inability to recreate the in vivo complexity of the biological environment, they are not suitable for accurate disease modeling. These limitations have been overcome by the development of three-dimensional (3D)-cell cultures systems [10]. The rationale design of 3D-cell cultures is to harvest cells in microstructures that resemble tissues or organs shape and organization, thus allowing better cell-to-cell/cell-environment contacts and signaling crosstalk [11,12,13,14,15], essential events for tissues correct development and functioning [14,15]. The 3D-cell culture strategy is articulated in many different methods and techniques that can be grouped in scaffold-based or non-scaffold based platforms, each with particular advantages and disadvantages that make them useful for different applications [10,16,17,18,19]. 3D-cell culture strategies are supported by the in silico modeling of multicellular systems that requires sophisticated techniques that go beyond the standard model formalisms in systems biology and allows complicated geometrical constructions [20,21]. In this regard, many computational tools allow predicting the more proficient cell model. These methods enable formulating both compartmental and spatial models with either 2D- or 3D-geometries, theoretically- or experimentally-derived. Several modeler environments have been developed in order to simulate and incorporate cell-based models, to describe the interactions and dynamics of cell-bound variables and to define molecular species in reaction–diffusion systems [22,23,24,25,26,27,28,29,30,31,32]. These computational tools allow simulating a wide range of cellular phenomena in space and time, both deterministically and stochastically (Table 1).

All of the above-mentioned techniques have been developed to respond to the compelling need to find better preclinical models for the study and modeling of disease pathogenesis.

## 3. Ex Vivo Stem Cell-Based Modeling Systems

Currently, stem cells (SCs) disease models represent the most powerful tool for having a better understanding of human disease pathogenesis and for developing new therapies [33]. As a matter of fact, the translation of stem cells intrinsic properties (i.e., self-renewal and pluri/multipotency) into an in vitro system allows the generation of selected cell lineages involved in the pathogenesis of a specific disease, along with promoting drug discovery, molecular screening, and transplantation therapies [33]. The first discoveries about SCs date back almost forty years when Evans and Kaufman isolated pluripotent cells from mouse embryos and established in vitro cultures of embryonic stem cells [34]. Since then, much research has been done in SCs field and today new stem cell-based disease models offer the possibility of personalizing the model according to the patient, observing a phenotype and then obtaining stem cells with the genotype of the donor subject, allowing to study also genetically complex disorders [35,36].

**Figure 1:**
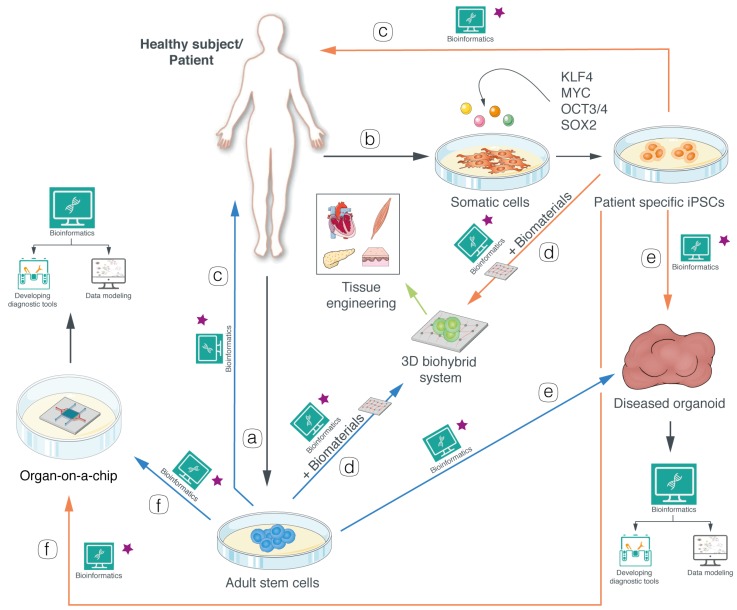
Schematic of ex vivo stem-cell based modeling systems. (**a**) Autologous stem cells from a donor heathy subject/patient, and patient-specific induced pluripotent stem cells (iPSCs) (**b**), may be source for generating: (**c**) Committed/differentiated cells; (**d**) 3D-biohybrid system; (**e**) organoids; and (**f**) organ-on-a-chip. The presence of computer icons indicates the critical role of the computational tools on the model design and the data elaboration. 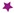, indicates the application of gene therapy and/or CRISPR/Cas9 to each system.

Among stem cell types, the most used for disease modeling purposes are adult stem cells (multipotent) [37] and induced pluripotent stem cells (iPSCs), because they permit the establishment of more advanced ex vivo models through: (i) Generation of committed/differentiated cell types involved in the disease; (ii) combination with biomaterials to generate a bio-hybrid system for tissue engineering applications; (iii) generation of organoids; and (iv) generation of organs-on-a-chip systems (Figure 1). Furthermore, the genome editing and gene therapy biotechnologies can be auxiliary tools to achieve the generation of more proficient stem cells disease models (Figure 1).

The advent of iPSCs opened a wide range of new stem cell applications. It is well-known that iPSCs can be derived from differentiated somatic cells, such as fibroblasts, by introducing defined transcriptional factors according to the Yamanaka and Takahashi protocol [38] and, like other pluripotent stem cells, have wide differentiation ability, giving rise to all cell derivatives of the three germ layers. As a matter of fact, the in vitro generation of iPSCs from somatic cells has made feasible the molecular study of different types of diseases, such as neurodegenerative diseases and cardiac diseases (see the list on Table 2), for which is difficult to obtain a tissue biopsy and autologous stem cells. For example, Israel et al. generated iPSCs from Sporadic Alzheimer’s disease patients, demonstrating that they have high levels of Amyloid-β and Tau protein hyperphosphorylation and that they represent a suitable model to study the correlation of phosphorylation of Tau, activation of GSK-3β and the processing of APP by the activity of β-secretase [39]. iPSCs have been also used to study the neuronal differentiation and the neurodegeneration in a familial form of amyotrophic lateral sclerosis [40] as well as to study the effect of α-synuclein gene triplication in Parkinson’s disease patients together with the identification of therapeutic compounds against α-synuclein accumulation [41].

Nevertheless, when somatic cells are reprogrammed to generate iPSCs, they acquire a series of new intrinsic properties that might generate hurdles in their applications. In fact, in addition to teratoma formation, karyotypic abnormalities and immature phenotype, they are characterized by unpredictable genetic background variations, thus making less reliable the corresponding disease model [42]. In this regard, genome editing tools enable creating isogenic controls iPSCs that differ from the original counterpart only on the gene/s responsible for the disease [43,44]. Despite these limitations, iPSCs have shown to be very promising for recapitulating complex genetically disorders especially when combined with gene editing techniques [33,45,46].

**Table 2:** List of human iPSCs disease models.

Disease Model	iPSC Source	Ref.
Adrenoleukodistrophy	Patient-derived fibroblasts	[47]
Alzheimer’s disease	Patient-derived fibroblasts	[39,48,49,50]
Amyotrophic lateral sclerosis	Patient-derived fibroblasts Patient-derived peripheral blood mononuclear cells	[40,51,52]
Becker’s muscular dystrophy	Patient-derived fibroblasts	[53]
Cardiovascular diseases	Patient-derived fibroblasts	[54,55,56]
Cockayne’s syndrome	Patient-derived fibroblasts	[57]
Down’s syndrome	Patient-derived fibroblasts	[53]
Duchenne muscular dystrophy	Patient-derived fibroblasts	[53,58]
Familial dysautonomia	Patient-derived fibroblasts	[59]
Fragile X-associated tremor/ataxia syndrome	Patient-derived fibroblasts	[60,61]
Gaucher’s disease type III	Patient-derived fibroblasts	[53]
Huntington’s disease	Patient-derived fibroblasts	[53,62,63,64]
Juvenile diabetes mellitus	Patient-derived fibroblasts	[53]
LEOPARD syndrome	Patient-derived fibroblasts	[65]
Machado-Joseph disease	Patient-derived fibroblasts	[66]
Lesch-Nyhan syndrome	Patient-derived fibroblasts	[53]
Parkinson’s disease	Patient-derived fibroblasts	[41,67]
Pompe’s disease	Patient-derived fibroblasts	[68]
Rett syndrome	Patient-derived fibroblasts	[69,70,71]
Prader-Willi syndrome	Patient-derived fibroblasts	[72,73]
Schizophrenia	Patient-derived fibroblasts	[74,75]
Spinal muscular atrophy	Patient-derived fibroblasts	[76]
Timothy syndrome	Patient-derived fibroblasts	[77,78]

The selective modification of cells’ genetic information was the groundbreaking discovery that revolutionized the concept of cell models. Innovative biotechnologies, together with other previously established, allowed the generation of pathological cells by introducing a defined mutation responsible for the onset of the disease (genome editing) as well as the restoration of a missing gene function in defective cells (gene therapy) (Figure 1). In the case of iPSCs or adult stem cells, they can afterwards be differentiated into diverse cell types involved in the pathology. Accordingly, it is plausible to be able to specifically study each pathological condition in each individual patient and consequently personalize the therapeutic approach (i.e., precision medicine) [79], also with the support of gene therapy approaches and gene editing procedures.

Briefly, gene therapy can be achieved through the use of advanced viral vectors (e.g., lentiviral, adeno-associated, adenoviral, retroviral [80,81,82,83,84,85]) or non-viral vector systems (e.g., liposomes systems) [86] that drive the therapeutic gene to the host cells [87,88,89,90,91]. The safety and efficacy of these strategies may be evaluated in engineered stem cell models, that are suitable for the treatment validation and for the screening of potential mutational insertions caused by the integration of the target gene in the host DNA [92,93,94,95]. Thus, even if the use of animal models is still essential before these treatments reach the clinical phase, stem cells are an alternative models enable to test the effectiveness of treatments in a highly efficient, reproducible and fast manner [84,85,86,90,91,96]. For instance, in Adenosine Deaminase Activity (ADA-SCID) immunodeficiency therapy (Strimvelis, GlaxoSmithKline (GSK), approved by EMA in 2016 [97]) ADA-genetically engineered hematopoietic stem cells served as basic cell model for validating the effectiveness of the gene delivery procedure and ultimately were used as therapeutic vehicle in vivo [97]. With the same end, in Metachromatic Leukodystrophy, hematopoietic stem cells transduced with the ARSA gene were useful for establishing the restoration of the biochemical metabolic defect and were also used as vehicle for the ARSA gene in patients affected [80,81]. Moreover, ongoing studies are showing the relevance of engineered stem cells (e.g., iPSCs, mesenchymal stem cells) for the expression of trophic factors for the treatment of neurodegenerative disorders (Parkinson’s disease, Alzheimer’s disease, Huntington’s disease, and amyotrophic lateral sclerosis [98]). Currently, many pathologies have been targets for gene therapy strategies and are being explored in clinical trials [81,99], or are already available for patients as they have obtained approval from the Food and Drug Administration (FDA) or the European Medicines Agency (EMA).

Genome editing can be achieved through the use of different nucleases that are able to introduce a double-strand break (DSB) in the double helix then resolved via non-homologous end joining (NHEJ) or homologous direct recombination (HDR). These molecular mechanisms are the bases of all the principal genome editing methods that, listed from the first used to the last discovered, are: Zinc finger nucleases (ZFNs, with specificity for 3 base pairs), transcription activator-like effector nucleases (TALENs, one base pair specificity) and the Clustered Regularly Interspaced Short Palindromic Repeat/Cas9 associated system (CRISPR/Cas9, single nucleotide specificity) [43,100]. While the first two approaches recognize the target sequence as a result of protein-DNA interactions, CRISPR/Cas9 system utilizes an RNA–DNA base pairing [100]. CRISPR/Cas9 is widely considered to be the most promising tool for manipulating gene expression because, compared to TALENs, it is 3–6 times more cost-effective, it is a much easier technique and in vivo delivery is more effective, although it shows less specificity due to its susceptibility to off-targets [101]. Specifically, CRISPR/Cas9 is engineered to edit DNA in stem cell models by designing specific guide RNA (gRNA) complementary to the target sequence (for extensive description see references [102,103,104,105,106] and Table 3 for computational tools for the design of gRNA). This technique allows manipulation of any cell genome for creating a specific cell phenotype useful to establish accurate ex vivo cell disease models, as well as for correcting DNA mutations linked to pathologies when combined with gene therapy procedures.

## 4. Ex Vivo Stem Cell-Based Systems: Bio-Hybrid Models for Tissue Engineering

Tissue engineering applies the concepts of engineering and biology to develop scaffold-based systems with the aim of reproducing the structure and the physiological functions of healthy tissues. The use of stem cells and biomaterials allows the generation of ad hoc bio-hybrid systems that represent 3D-models of tissues either for disease modeling (Figure 1) or, after validation, for therapeutic transplantation [14,37,113,114].

These scaffold-based platforms rely on the mechanobiology concept by which biomaterials provide the 3D-structure of native tissues and through their chemical/physical properties are able to specifically steer stem cells toward tissue-specific phenotypes [14]. As a matter of fact, the modulation of physical and chemical properties of biomaterials such as architecture, shape, mechanical properties, and surface structure, have been showed to control the biological responses of stem cells (both stemness and differentiation properties) [113,115,116]. Many types of natural or synthetic biomaterials, such as polymers, ceramics, bio-glass, composites, or seldom metals [37,117,118,119,120,121,122,123], have been combined with different types of stem cells in order to faithfully recapitulate key environmental signals (thus allowing finer cell/tissue models [11,124]) and elucidate complex biological phenomena such as stem cell reprogramming pathways and determination processes [14]. To date, some tissue engineering strategies have already reached the market and are used as personalized therapies. (Table 4) [125,126]. For example, many types of skin substitutes, acellular or cellular, are currently available for the treatment of wounds, while both cryopreserved placental membrane and human fibroblasts-derived substitute (HFDs) are approved for the therapy of diabetic foot ulcers [127] (Table 4). Moreover, autologous cartilage cells are used in a phase III clinical trial for the treatment of articular cartilage defects [128] (Table 4).

Among the many scaffold fabrication methods, 3D-bioprinting has recently received a great deal of attention as one of the best tool to obtain specific precise architecture and biochemical composition of biocompatible materials and supporting components [135]. With this technique it could be possible to faithfully recreate native tissue cellular composition, vasculature, and architecture in vitro, thus making biomimetic tissue models, which could then be used for studying disease mechanisms, drug screening, and clinical applications [136,137].

Accordingly, 3D-bioprinting was used for generating in vitro disease models of tumor microenvironments [138], ovarian cancer [139], and drug testing [140]. Even though bioprinting of tissues and organs gave new possibilities to tissue engineering and regenerative medicine [136], their translational application is still facing some challenges. As a matter of fact, current obstacles in 3D-bioprinting methods are the poor scalability, the improvement of bioinks with appropriate biological and mechanical properties suitable to different cell types and the absence of vascularization that cause hurdles in nutrients supply, other than regulatory aspects [141]. Nevertheless, research in 3D-bioprinting technology is advancing and these limitations will be overcome [141].

## 5. Ex Vivo Stem Cell-Based Systems: Organoids

In recent years, progress in 3D-stem cell cultures led to the production of new systems with great potential as cell model platforms (Figure 1). These models have been called organoids, because of their ability to resemble organs, and are characterized by (i) 3D-structure, (ii) different types of cells normally present in the organ that should be modelled, (iii) functional features of the organ, and (iv) self-organization [142]. Self-organization is indeed the most important characteristic of organoids: Under opportune culture conditions (appropriate nutrients and growth factors) primary cells isolated from biopsies, embryonic or adult stem cells, and patient-derived iPSCs can go towards differentiation to develop a ‘mini’ organ according to intrinsic developmental patterns, thus generating a faithful replica of tissues’ morphology and organization [142,143]. Therefore, organoids hold a great promise for ex vivo cell modeling as they are a powerful tool for developmental studies and personalized medicine [144]. Accordingly, organoids represent a major improvement in disease modeling compared to animal models, as they overcome ethical issues, are more cost-effective and allow faster analysis [144,145]. Since the development of the first intestinal organoids by Sato and collaborators in 2009 [146], many other models of various tissues have been developed following diverse approaches such as (i) Matrigel scaffold-based, (ii) Embryoid Bodies (EB)-based, and (iii) Air–Liquid Interface (ALI) method [147], and they have been used to model a great range of pathologies, from viral infection to solid cancers, cystic fibrosis, and endometriosis (Table 5). Even if in the last decade efforts have been made for improving organoids fidelity to the in vivo counterpart [148], there are still several critical aspects that have to be taken into account when evaluating organoids predictability. In particular, there is poor control on the shape, size and cells appropriate organization, causing hurdles in terms of scalability and high-throughput approaches. All these variables are mainly arising from a lack of nutrients supply due to the absence of physiological vascularization, which causes the inner part of the organoid to suffer metabolic stress and go towards incorrect differentiation or death [142,143,149]. Although these limitations are slowing down organoids’ clinical applications, they are still considered one of the best options currently available to build a strong cell model capable of summarizing key features of many diseases.

## 6. Ex Vivo Stem Cell-Based Systems: Organs-on-a-Chip

A more recent advance in stem cell biology and 3D-tissue engineering is the innovative application of microfluidic techniques for the development of organ-on-a-chip platforms (OOC) (Figure 1). The rationale of the introduction of microfluidic in cell cultures is to reproduce the microenvironment of cells through the use of precise control on fluid flow, biochemical factors and mechanical forces [198]. The aim of OOCs is to reproduce in vitro functional units of organs by reproducing the essential elements that allow physiological functions [199]. This is achieved by the use of micro-fabricated cell culture devices designed to replicate the fundamental architectural characteristics of the organ in exam, which incorporate microchambers and microchannels that allow the growth of diverse cell types in defined culture condition thanks to the capillary controlled fluid flow. Moreover, the tailor-made architectural organization of OOCs enables to study the interactions between different biological compartments, such as cells and the extracellular matrix (ECM), tissue–tissue interfaces and parenchymal-vascular association [199,200]. One of the most important aspects of OOCs is that it is possible to combine different biomaterials, microfabrication techniques (extensively reviewed in [201,202]) and cell types for creating multi-compartment and multiphysiological systems that can model tissues pathophysiology. These systems can be developed for reflecting individual pathophysiological conditions by including blood samples, patient-derived primary adult stem cells or iPSCs and by adjusting physiochemical parameters of the flow according to personal health data [203] (Figure 1). This personalized strategy could thus be the new frontier for building a tailored cell disease model able to take into account individual pathological variability and, in this way, personalizing treatments [203]. The possibility of harnessing stem cells versatility, differentiated cells specific properties and microfluidic control allowed to build disease models with unprecedented features, as it made possible to reproduce in vitro complex biological structures that could not be obtained with previous cell culturing technologies such as the blood–brain barrier [204] (Table 6). As a matter of fact, in the past five years many disease models have been developed, such as lung-on-a-chip for cancer [205] or coupled-OOCs of liver and pancreas spheroids able to maintain glucose homeostasis for modeling type 2 diabetes [206] (Table 6). Of note, different OOC models can be linked to build an ideal ‘human-on-a-chip’ which could theoretically serve as the ultimate alternative to animal models for its capacity to predict multiorgan biological interactions and response to therapeutic treatments [202,207].

In spite of the many advantages that OOCs have, they rely on the ability of the scientist to artificially reconstruct the correct microenvironment using the correct cell types and arranging them as they would be in the physiological state. Therefore, future perspective of 3D cell modeling will involve the synergic combination of OOCs and organoids, for the possibility of precisely control microenvironmental conditions and exploit organoids self-organization, with the aim of obtaining the best ex vivo cell modeling technology [208].

**Table 6:** List of human organ-on-a-chip disease models.

Organ	Disease	Model Derivation	Ref.
BRAIN	Alzheimer’s disease	Commercial neural progenitor cells and commercial microglia cell line	[204,209,210,211,212]
	Blood–brain barrier dysfunctions	Commercial cell lines (endothelial cells, brain pericytes, astrocytes) and healthy donors-derived iPSCs	
	Neuroinflammation	Commercial cell lines (endothelial cells, brain pericytes, astrocytes)	
	Brain cancer	Commercial glioblastoma cells	
HEART	Mitochondrial cardiomyopathy of Barth syndrome	Patients-derived iPSCs	[213,214,215]
	Chronic drug exposure	Commercial human embryonic stem cells	
KIDNEY	Antibiotic nephrotoxicity	Healthy donors human kidney tissues	[216]
LIVER	Hepatitis B infection	Commercial HepDE19 cells, Primary human hepatocytes, Kupffer Cells, HepG2 cells	[217,218,219,220,221]
	Drug hepatotoxicity	Commercial HepG2 cells, human umbilical vein cells (EAhy926), human stellate cells (LX-2), human histiocytic lymphoma (U937) and basement membrane extract	
	Drug hepatotoxicity	Commercial primary human hepatocytes, monoblast derived Kupfer cells, stellate cells (LX-2), human dermal microvascular endothelial cells and isolated primary human polymorphonuclear leukocytes from healthy donors	
	Non-alcoholic fatty liver disease	Commercial HepG2 cells	
LUNG	Protein-induced lung inflammation	Commercial bronchial epithelial cell line and healthy donors fibrocytes	[205,222,223]
	Idiopathic pulmonary fibrosis	Commercial alveolar epithelial-like cells	
	Lung cancer	Commercial non-small cell lung cancer cells and commercial human fetal lung fibroblasts	

## 7. Conclusions

In this review, we have documented the significant improvements in disease modeling using human adult stem cells and iPSCs, highlighting how their contribution in generating more elaborated 3D-models (organoids, organ-on-a-chip, and bio-hybrid systems) have greatly facilitated our understanding and ability to treat human disease. Noteworthy, we have also pointed out that the tremendous advancement of the field has highlighted the need for these models to be accurate, efficient and safe, in order to accomplish valid differentiation protocols and development of effective therapies. In this regard, computational tools able to predict complex biological systems, together with genome editing and gene therapy technologies support researchers in the development of more proficient disease models. Collectively, the issues discussed emphasized ex vivo-stem cell models as an alternative or complementary system to animal disease models.

## Figures and Tables

**Table 1:** List of modeler environments software.

Software	Application	Link	Ref.
BIOCHAM4	A software environment for modeling biochemical systems	https://lifeware.inria.fr/biocham4/	[22]
Bio-SPICE	Biological simulation software for intra- and inter-cellular evaluation, modeling and simulation of spatio-temporal processes in living cells	http://biospice.sourceforge.net	[23]
CellML	CellML allows scientists to store and share computer-based mathematical models	https://www.cellml.org	[24]
COPASI: Biochemical System Simulator	A software application for simulation and analysis of biochemical networks and their dynamics	http://copasi.org	[25]
E-Cell System	A software platform for modeling, simulation and analysis of complex, heterogeneous and multi-scale systems like the cell	http://www.e-cell.org	[26]
EPISIM	A platform for graphical multi-scale modeling and simulation of multicellular systems	http://tigacenter.bioquant.uni-heidelberg.de/episim.html	[27]
MCell	A program that uses spatially realistic 3D cellular models and specialized Monte Carlo algorithms to simulate the movements and reactions of molecules within and between cells	http://www.mcell.psc.edu	[28,29]
Morpheus	A modeling and simulation environment for the study of multi-scale and multicellular systems	https://morpheus.gitlab.io	[30]
Tissue Simulation Toolkit	A two-dimensional library for the Cellular Potts Model to study tissue patterning and developmental mechanisms	https://biomodel.project.cwi.nl/software/software#TST	[31]
VCell	A general computational framework for modeling physicochemical and electrophysiological processes in living cells and for treating spatially resolved models	https://vcell.org	[32]

**Table 3:** List of on-line tools for guide RNA (gRNA) design.

Software	Link	Ref.
CasFinder	http://arep.med.harvard.edu/CasFinder/	[107]
CHOP-CHOP	https://bitbucket.org/valenlab/chopchop/src/master/	[108]
CRISPR-ERA	http://crispr-era.stanford.edu/	[109]
CRISPR-DO	https://bitbucket.org/jianma/crisprdo/src/default/	[110]
GuideScan	https://bitbucket.org/arp2012/guidescan_public/src/master/	[111]
mm10db	https://github.com/bmds-lab/mm10db/	[112]

**Table 4:** List of tissue engineering therapeutic approaches used in clinic.

Product	Pathology	Approval	Company	Ref.
ChondroCelect	Cartilage diseases	European Medicines Agency (EMA) (2009, now withdrawn)	TiGenix.	[126]
Dermagraft	Diabetic foot ulcer and venous leg ulcer	USA FDA (2010)	Organogenesis inc.	[127]
Epicel	Deep dermal or full-thickness burns	USA FDA (2016)	Vericel Corporation	[129,130]
Holoclar	Limbal stem cell deficiency	EMA (2015)	Chiesi Farmaceutici S.p.A.	[131]
Maci	Knee cartilage lesions	USA FDA (2016) EMA (2013, now withdrawn)	Vericel Denmark ApS	[132,133]
Spherox	Cartilage defects in knee joints	EMA (2017)	Codon AG	[134]

**Table 5:** List of human organoids disease models.

Organ	Disease	Model Derivation	Ref.
Bladder	Bladder cancer	Patients tumor biopsies	[150,151]
Blood vessel	Diabetic vasculopathy	Commercial iPSCs and patient-derived endothelial cells	[152]
Brain	Microcephaly	Commercial ESCs and commercial iPSCs.	[153,154,155,156,157,158,159,160,161,162,163,164,165,166,167]
	Autism spectrum disorder (ASD)	iPSCs derived from idiopathic ASD families	
	Miller-Dieker syndrome (lissencephaly)	Patient-derived iPSCs	
	Glioblastoma	Patients tumor biopsies	
	Neonatal microcephaly due to Zika virus infection	hiPSCs and human embryonic stem cells (hESCs)	
	Schizophrenia	Patient-derived iPSCs	
	Familial Alzheimer’s disease	Patient-derived iPSCs	
	Parkinson’s disease	Patient-derived iPSCs	
Breast	Breast cancer	Patients tumor biopsies	[168]
Colon	Cancer	Patients tumor biopsies	[169]
Endometrium	Endometriosis	Patients tumor biopsies	[170,171]
	Endometrial cancer	Patients tumor biopsies	
	Lynch syndrome	Patients tumor biopsies	
Esophagus	Esophageal Adenocarcinoma	Patients tumor biopsies	[172]
Heart	Local Injury	Commercial hESCs	[173,174,175]
Intestine	Cystic fibrosis (CF)	Intestinal biopsies and crypt isolation	[176,177,178,179,180]
	Acute gastroenteritis due to human Noroviruse infection	Healthy donors’ intestinal biopsies	
	Diarrheal illness due to human Rotavirus infection	Healthy donors’ duodenal and ileal biopsies	
	Diarrheal illness due to *Cryptosporidium* infection	Healthy donors’ duodenal biopsies	
	Respiratory infection due to Middle East respiratory syndrome coronavirus	Healthy donors’ colon biopsies	
Kidney	Nephronophthisis	Patients derived iPSCs	[181]
LIVER	α1-antitrypsin deficiency	Liver biopsies	[182,183,184,185]
	Primary liver cancers	Patients tumor biopsies	
	Hepatitis B infection	Healthy donor iPSCs	
	Hepatitis E infection	Liver biopsies of patients affected	
LUNG	Lung cancer	Non-small cell lung cancer biopsies	[179,186,187,188]
	Diarrheal illness due to *Cryptosporidium* infection	Non-small cell lung cancer biopsies	
	Influenza virus infection	Healthy donor’s lung biopsies	
	Lung bronchiolitis and fibrosis due to respiratory syncytial virus infection	hPSCs	
PANCREAS	Pancreatic ductal adenocarcinoma	Patients tumor biopsies	[189,190,191]
PROSTATE	Prostate cancer	Patients metastasis samples	[192]
RETINA	Leber congenital amaurosis	Patient-derived iPSCs	[193]
STOMACH	Gastric cancer	Patients tumor biopsies	[194,195,196,197]
	Gastric diseases due to *Helicobacter pylori* infection	Gastric/esophageal tumor biopsies or commercial PSCs	
